# The Impact of SARS-CoV-2 Pandemic on the New Cases of T1DM in Children. A Single-Centre Cohort Study

**DOI:** 10.3390/jpm11060551

**Published:** 2021-06-13

**Authors:** Anca Andreea Boboc, Carmen Nicoleta Novac, Maria Teodora Ilie, Mara Ioana Ieșanu, Felicia Galoș, Mihaela Bălgrădean, Elena Camelia Berghea, Marcela Daniela Ionescu

**Affiliations:** 1 Marie Curie Emergency Children’s Hospital, 041451 Bucharest, Romania; anca.orzan@umfcd.ro (A.A.B.); dr.carmen.novac@gmail.com (C.N.N.); maria-teodora.ilie@rez.umfcd.ro (M.T.I.); mara-ioana.iesanu@rez.umfcd.ro (M.I.I.); felicia.galos@umfcd.ro (F.G.); mihaela.balgradean@umfcd.ro (M.B.); daniela.ionescu@umfcd.ro (M.D.I.); 2 Department of Pediatrics, Carol Davila University of Medicine and Pharmacy, 020021 Bucharest, Romania

**Keywords:** early diagnosis, type 1 diabetes mellitus, children, COVID-19, epidemics, diabetic ketoacidosis

## Abstract

Type 1 diabetes mellitus (T1DM) represents one of the most frequent chronic illnesses affecting children. The early diagnosis of this disease is crucial, as it plays a key role in preventing the development of a life-threatening acute complication: diabetic ketoacidosis. The etiopathogenetic role of viral infections has long been suggested and emerging data are pointing towards a complex bidirectional relationship between diabetes and COVID-19. The aim of this study is to assess the impact of the COVID-19 pandemic on the incidence and severity of new T1DM cases in children in Romania. We analyzed the differences between a group of 312 patients diagnosed with T1DM in the period 2003–2019 and a group of 147 children diagnosed during the pandemic. The data were investigated using statistical analysis of a series of relevant variables. The total number of newly diagnosed T1DM increased by 30.08% in the period March 2020–February 2021 compared to the previous years. The patients in the pandemic group had a higher mean age at the onset of T1DM, were less frequently living in an urban area, and presented a higher mean value of HbA1c. Diabetic ketoacidosis at the onset of T1DM was 67.40% more frequent, and a higher percentage of these patients presented with a severe form. The duration of T1DM symptoms did not differ significantly between the two groups. A number of 8 patients associated SARS-CoV-2 infection at the time of T1DM diagnosis.

## 1. Introduction

Type 1 diabetes mellitus (T1DM) is a metabolic disease characterized by the autoimmune-mediated destruction of the pancreatic β-cells that leads to a deficit in the production of insulin with various repercussions on the intermediary metabolism. T1DM represents one of the most frequent chronic illnesses in pediatric population and has shown a continuously increasing incidence over the last decades [1]. The most frequent clinical manifestations of T1DM are weight loss, polyuria, and polydipsia; therefore, the rapid recognition of these symptoms plays a key role in making an early diagnosis and thus avoiding the progression towards diabetic ketoacidosis (DKA). DKA is a life-threatening acute complication that can be associated with the onset of T1DM. The incidence of DKA varies widely from one geographic region to another, ranging from 15% to 70% in Europe and North America [2].

The specific etiopathogenetic mechanisms involved in the development of T1DM are not completely understood so far, but they are known to entail a complex interaction between the genome, metabolic processes, immune system characteristics, environmental factors, and microbiome [3]. Among the environmental factors, viral infections have long been considered triggers of the autoimmune process leading to the development of T1DM. Various mechanisms could explain the role that viral infections play in promoting the onset of T1DM in children. In patients with a predisposing background of genetic and immunological factors, the exposure to certain viruses might accelerate the final processes leading to the onset of the disease [4].

COVID-19 is a highly infectious respiratory disease determined by SARS-CoV-2 that spread rapidly across the world, having been declared a pandemic on 11 March 2020. In children, the disease has a milder course, being asymptomatic in many cases and having a mortality rate of 0.18% in patients hospitalized for COVID-19 [5]. In Romania, the first case of SARS-CoV-2 infection was reported on 26 February 2020. On 28 February 2021, the total number of confirmed cases exceeded 800,000 (41.77 per 1 million people, a prevalence that made the country rank 24th in the world by the number of infections). Over 40,000 (5%) of these infections were reported among children [6].

Although extensive evidence has been gathered since the beginning of the COVID-19 pandemic about the complex bidirectional relation between SARS-CoV-2 infection and diabetes, data regarding T1DM specifically remain sparse. A multicentre study reported an 80% increase in new T1DM cases in children, some of these patients having a confirmed history of SARS-CoV-2 infection [7]. Several studies reported a significant increase in the frequency of DKA present at the diagnosis of T1DM, with a higher percentage of the severe form of this metabolic complication [7,8,9,10,11,12]. It was hypothesized that an explanation for this phenomenon is the delay in the presentation of these patients caused by the parents’ fear of accessing the healthcare system [9,10]. However, reports from the United Kingdom showed a short duration of symptoms in the majority of children newly diagnosed with T1DM [7]. More information about the complex interactions between T1DM and COVID-19 was gathered due to a study which showed that SARS-CoV-2 is able to infect cultured human pancreatic islets and replicate inside these cells, altering the subcellular morphology and the response to glycemic variations [13]. The pancreatic endocrine cells strongly express the angiotensin converting enzyme 2 (ACE2) receptor, a main binding site for SARS-CoV-2 [14].

Another possible mechanism that could play a role in the observed increased number of patients with T1DM and other autoimmune disorders during the pandemic could be the decreased vitamin D levels, a possible consequence of strict lockdown measures with people spending significantly less time outside. Vitamin D, due to its immunomodulatory properties, shows multiple beneficial effects in T1DM [15]. This idea is supported by a study reporting that 2000 UI cholecalciferol per day reduced T1DM incidence by 78% [16]. Moreover, the decision of the Finish authorities to fortify dietary milk products with vitamin D had led to a decrease in the number of T1DM cases [16]. From another point of view, the capacity of vitamin D to enhance the immune system might ensure a protective effect from developing severe forms of SARS-CoV-2 infection and reduce the risk of contracting the disease [17].

The aims of this study were to assess a possible increase in the number of T1DM new cases in children from Bucharest and the surrounding areas during the COVID-19 pandemic, compared to previous years, to analyze the differences in patient and disease characteristics between the pre-pandemic and pandemic periods and to evaluate predictors (including presentation time) of DKA associated with the onset of the disease and DKA severity.

## 2. Materials and Methods

### 2.1. Study Population

We performed an observational retrospective cohort study that included pediatric patients with T1DM from Marie Curie Emergency Children’s Hospital, Bucharest. Our institution is a reference centre for pediatric T1DM, all the new cases from Bucharest and the south-east region of Romania (approximately one quarter of the 3,895,000 children in Romania are living here) being managed here. In order to analyze the temporary evolution of newly diagnosed pediatric T1DM cases, we extracted from the electronic hospital registry the numbers of all new T1DM cases diagnosed in patients younger than 18 years old, for each month of 2018, 2019, and 2020. Patients with other types of diabetes were excluded.

In order to further assess patients and disease differences between children diagnosed during (March 2020–February 2021; pandemic group) and before the COVID-19 pandemic (2003–2019; pre-pandemic group), we split the study sample into 2 groups. The pre-pandemic group included patients under 18 years old, diagnosed with T1DM, with extensive follow-up and complete and easily available medical records. The pandemic group included all the children diagnosed with T1DM in the period mentioned before; thus, the follow-up criteria being impossible to apply considering the recent onset.

### 2.2. Patients and Disease Characteristics

The data were collected from the digital and paper-based medical records of the hospital. For each patient, we gathered the following information: age at the onset of T1DM, gender, living area, glycosylated hemoglobin (HbA1c) levels, pH, and duration of T1DM specific symptomatology. The diagnosis of T1DM was made on the following criteria: fasting plasma glucose levels higher than 126 mg/dL or symptoms of hyperglycemia (polyuria, polydipsia, unexplained weight loss) with a random plasma glucose level ≥200 mg/dL or 2-h plasma glucose ≥200 mg/dL during an oral glucose tolerance test.

The pH was determined at the time of presentation, and a level lower than 7.3 was defined as DKA. The degree of severity of DKA was classified according to 2018 International Society for Paediatric and Adolescent Diabetes (ISPAD) guidelines: mild DKA (pH < 7.3), moderate DKA (pH < 7.2), severe DKA (pH < 7.1).

HbA1c was measured through an immunoturbidimetric method (DCCT standardized: Diabetes Control and Complications Trial and NGSP certificated: National Glycohemoglobin Standardization Program). The normal levels were 4.8–6.4%.

### 2.3. Statistical Analyses

Patients and disease characteristics were reported as absolute and relative frequencies for categorical variables, medians, and interquartile ranges (IQR) for non-normally distributed continuous variables and means and standard deviations for normally distributed continuous variables. Tests of normality were conducted with the Kolmogorov-Smirnov and Shapiro-Wilks tests. The variables were reported for the overall study sample, and separately, according to the time of diagnosis (pre-pandemic/pandemic group), according to DKA presence and DKA severity. Differences between the groups were tested using χ^2^ statistics for categorical variables, Wilcoxon or Kruskal-Wallis rank sum tests for non-normally distributed continuous variables, and *t*-tests or ANOVA for normally distributed continuous variables. The eight cases that presented SARS-CoV-2 infection at the time of T1DM diagnosis were described separately based on multiple variables.

Logistic regression was performed to determine the effects of patient characteristics and time of presentation (independent variables) on the likelihood of DKA being present at the time of T1DM diagnosis (dependent variable). Proportional odds logistic regression was performed to assess effects on the likelihood of increased DKA severity (3-level ordered variable) for the subset of patients presenting DKA. Univariable and multivariable analyses were conducted for both outcomes. Patients’ characteristics were included in multivariable analysis, regardless of their significance in univariable analysis, in order to estimate the independent effect of time of presentation on the outcomes.

Furthermore, to address selection bias induced by the selection of patients in the pre-pandemic group, the analyses were repeated on a propensity score-matched dataset. Propensity scores for being diagnosed during or before the pandemic period were estimated with logistic regression, using all baseline measured characteristics. Patients were matched using 1:1 greedy nearest neighbor matching without replacement. The balance of individual characteristics between the pandemic and pre-pandemic groups, before and after matching, was assessed using standardized mean differences (SMD). In the matched dataset, containing patients with similar characteristics, the effects of time of presentation (pre-pandemic or pandemic) on presence of DKA and DKA severity were estimated. Residual differences in observed characteristics between the pre-pandemic and pandemic groups were accounted for by covariate adjustment.

Restricted cubic splines were used to test for non-linear relationships between continuous independent variables (age, HbA1c) and the outcomes of interest in the model development phase. The estimated associations were reported as odds ratios (OR) and 95% confidence intervals (95% CI). To assess if the predictive strength of multivariable models was increased by adding time of presentation as a covariate, the proportion of explained outcome variability was calculated using model Nagelkerke’s pseudo-R^2^.

The percentage of missing values for each variable was reported in the descriptive tables and regression analyses were conducted on complete cases.

The data were statistically analyzed using Microsoft Excel (Microsoft Corp. S.R.L, MI, USA), GraphPad Prism? (version 6.0; GraphPad Prism Inc., CA, USA), R (R version 4.0.3; R Foundation for Statistical Computing, Vienna, Austria) and R Studio (version 1.1.463; R Studio, Inc., Boston, MA, USA) considering statistical significance at a two-sided *p*-value of 0.05. Nearest neighbor matching was performed using the MatchIt package in R.

## 3. Results

### 3.1. Analysis of Monthly New Type 1 Diabetes Cases

**Figure 1:**
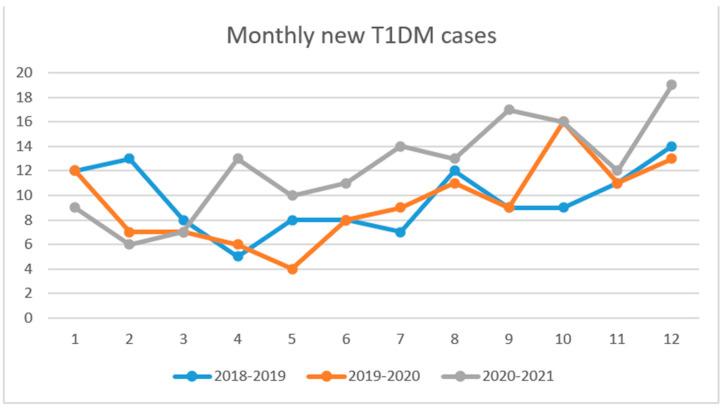
The number of new T1DM cases per month for the periods March 2018–February 2019, March 2019–February 2020 and March 2020–February 2021.

In March and April 2020 the number of new T1DM cases was lower than the same period of 2018 and 2019. Between May 2020 and February 2021 the monthly number of new T1DM cases was higher, with a mean of 13.2 cases/month compared to the same period of the last two years with a mean of 9.4 cases/month (*p*-value = 0.015). The total number of newly diagnosed T1DM increased by 30.08% from 113, between March 2019 and February 2020, to 147 cases reported between March 2020 and February 2021 (Figure 1).

### 3.2. Demographic Analysis

The study included a number of 459 pediatric patients with a mean age at diagnosis of 7.59 years, the age group with the highest percentage of patients being 8–11 years (32.68%). The male/female ratio was 53%/47% (245/214), the background was urban in 352 cases (77%), and rural in 107 (23%).

### 3.3. Differences between the Pre-Pandemic and the Pandemic Group of Patients

**Figure 2:**
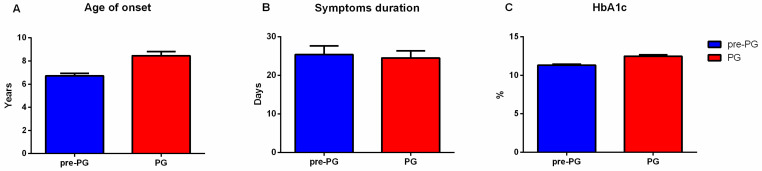
Comparison between the pre-pandemic (pre-PG) and pandemic group(PG) of patients based on several parameters. (**A**) Age of onset; (**B**) Symptoms duration; (**C**) HbA1c.

The patients in the pandemic group had a higher median age at the onset of T1DM than those diagnosed during the period 2003–2019 (7.20 [7.07, 7.30] years vs. 7.00 [3.00, 10.00] years, *p* < 0.001). The sex ratio was lower in the pandemic group compared to the pre-pandemic group (M/F = 1.19 and 1.04, respectively, *p* = 0.55). The percentage of patients living in an urban area decreased from 83.65% before 2020 to 61.90% during the pandemic (*p*-value < 0.001). There was no significant difference in the mean duration of T1DM symptoms before presentation between the two groups (25.39 ± 2.23 days vs. 24.52 ± 1.83 days, *p* = 0.51). In the pandemic group the values of HbA1c were higher than those of the patients from the pre-pandemic group (mean HbA1C 12.47 ± 2.19% vs. 11.32 ± 2.18%, *p* < 0.001) (Table 1, Figure 2). In the pandemic group, one patient was diagnosed with both autoimmune thyroiditis and celiac disease at the time of T1DM diagnosis.

**Figure 3:**
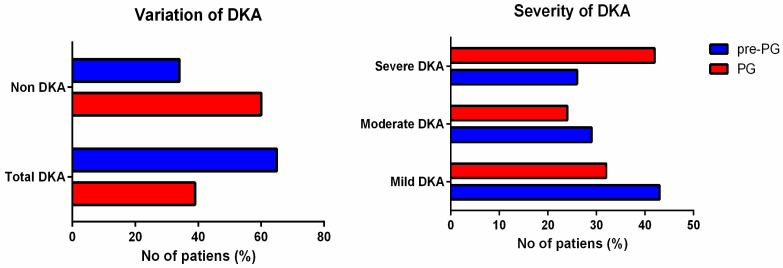
DKA presence and severity in the pre-pandemic group of patients (pre-PG) and the pandemic group (PG).

The proportion of DKA at the onset of the disease increased during the pandemic with 67.40%, from 39.42% in the pre-pandemic group to 65.99% in the pandemic group (OR = 2.98, CI 95% = 1.97–4.49, *p* < 0.0001). In the pandemic group, a higher percent of DKA cases developed the severe form compared to the pre-pandemic group (42.27% vs. 26.83%, OR = 1.99, CI 95% = 1.13–3.51, *p* = 0.016). (Table 2, Figure 3).

### 3.4. Association of Patient Characteristics with DKA Diagnosis and Severity

In order to further assess the development of DKA the sample was divided into two groups based on the presence and absence of DKA at the time of diagnosis. The gender distribution and the background distribution did not differ significantly between the two groups. 

The median age was lower in the group of patients that presented with DKA (7.00 [3.00, 10.00] vs. 8.00 [5.00, 11.00], *p* = 0.006). Also, in this group HbA1c had a higher mean value (11.96 ± 2.19 vs. 11.45 ± 2.27), and a significantly higher percent of patients were diagnosed during the pandemic (44.1% vs. 20.9%) (Table 3).

The group of patients that presented DKA at the time of diagnosis was further divided into three subgroups based on DKA severity. Gender distribution, the median age at diagnosis, background distribution, mean HbA1c, and the time of diagnosis (i.e., pre-pandemic/pandemic) did not differ significantly between the two groups (Table 4).

In univariable analysis, a significant association with a higher likelihood of being diagnosed with DKA was found for younger age (OR 0.94 per year increase in age, 95% CI 0.90–0.98), higher levels of HbA1c (OR 1.11 per percent increase in HbA1c, 95% CI 1.02–1.21) and presentation during COVID-19 pandemic (OR 2.98, 95% CI 1.99–4.52 compared to the pre-pandemic period). All three variables remained significantly associated with the likelihood of DKA in multivariable analysis, with the same direction of effects and larger effect estimates: OR 0.87 per year increase in age (95% CI 0.82–0.92), OR 1.17 per percent increase in HbA1c (95% CI 1.06–1.30) and OR 3.23 for presentation during the pandemic, compared to the pre-pandemic period (95% CI 2.06–5.15). Gender and urban/rural background were not significantly associated with the likelihood of being diagnosed with DKA in our sample. Multivariable model Nagelkerke’s pseudo-R2 for DKA diagnosis was 8% without time of presentation and 15% with time of presentation (Table 5). In sensitivity analysis (*n* = 439), excluding patients with active SARS-CoV 2 infection at the time of T1DM diagnosis, the effects of the variables remained similar in both magnitude and significance: OR 3.15 for presentation during the pandemic, compared to the pre-pandemic period (95% CI 2.00–5.05) in multivariable analysis.

In the subgroup of patients with DKA, the only variable that demonstrated a significant association with increased DKA severity was presentation during the pandemic (OR 1.85 compared to the pre-corona period, 95% CI 1.07–3.23 in multivariable analysis). Multivariable model Nagelkerke’s pseudo-R2 for DKA severity was 1% without time of presentation and 4% with time of presentation. (Table 6). In sensitivity analysis (*n* = 212), excluding patients with active SARS-CoV 2 infection at the time of T1DM diagnosis, presentation during the pandemic was similarly the only variable to display a significant effect on increased DKA severity: OR 1.99, compared to the pre-pandemic period (95% CI 1.13–3.51) in multivariable analysis.

The cohort of propensity score-matched patients included 294 patients and consisted of all the patients in the pandemic group and their matched “controls” from the pre-pandemic group. The balance of individual characteristics between the groups, before and after matching, is presented in Supplementary Table S1 and Supplementary Figure S1.

The positive effect of presentation during the pandemic on presence of DKA at the time of T1DM diagnosis was significant in the matched cohort, in both univariable analysis (OR 3.2 compared to the pre-pandemic period, 95% CI 2–5.2, *p* < 0.001) and multivariable analysis with covariate adjustment (OR 3.4 compared to the pre-pandemic period, 95% CI 2.1–5.6, *p* < 0.001).

In the subset of 152 patients with DKA from the matched cohort, presentation during the pandemic was significantly associated with increased DKA severity, in both univariable analysis (OR 2.03 compared to the pre-pandemic period, 95% CI 1.1–3.77, *p* = 0.024) and multivariable analysis with covariate adjustment (OR 2.03 compared to the pre-pandemic period, 95% CI 1.09–3.82, *p* = 0.027).

### 3.5. SARS-CoV-2 Infection at the Onset of Type 1 Diabetes Mellitus

Eight of the patients diagnosed with T1DM in 2020 presented SARS-CoV-2 infection at the same time. The diagnosis of COVID was made on the positive results of the RT-PCR for SARS-CoV-2 tests. These patients’ ages ranged from 1 year to 17 years, with a mean value of 8.87 and the male/female ratio was 1/1. None of the patients presented any significant past medical history. Regarding the presence of type 1 diabetes or other autoimmune diseases in the family medical history, two patients’ mothers were diagnosed with autoimmune thyroiditis and one patient’s cousin was diagnosed with T1DM. In half of these cases, the duration of T1DM characteristic symptoms (polyuria, polyphagia, weight loss) was 14 days; in two cases, it was 7 days; one patient had a 60 days history of the specific clinical manifestations, and one other patient presented no symptoms before the diagnosis. Among the eight patients, seven presented DKA which was mild in three cases, moderate in three, and severe in one patient. The HbA1c had a mean value of 12.19%, with a minimum of 10.70% and a maximum of 13.80%. The COVID-19 infection was completely asymptomatic in four of the children, one patient aged 2 presented fever (maximum temperature = 40 °C), eating refusal, and somnolence, two patients experienced vomiting, and abdominal pain (that could have been determined by SARS-CoV-2 infection, as well as by DKA) and one patient was diagnosed with stomatitis and streptococcal pharyngitis. The presence of inflammation based on an increased level of C reactive protein was detected in five patients, with slightly elevated levels in three patients and a maximum value of 39.98 mg/dL in the patient that presented fever. Vitamin D deficiency was identified in two of the five measurements performed (Table 7).

## 4. Discussion

Given the paucity of available information on the subsiding complex relations between SARS-CoV-2 infection and other illnesses, it is important to explore the possible effects of the pandemic on the incidence, severity, pattern, and evolution of the existing conditions in order to adjust the healthcare response to the present situation and to anticipate what the future may hold. Taking that into consideration, this article assessed the evolution of the number and severity of new T1DM cases during the pandemic.

Between March 2020 and February 2021 there was an increase of 30.08% in T1DM cases compared to the same period of the previous year (147 vs. 113). This observation is in agreement to another study showing an increase in new T1DM cases in children in 2020 [7]. It is important to mention that the changes in the functioning and organization of the healthcare system as a response to the pandemic could not have contributed significantly to the increase in the number of new cases, because of the fact that the majority of new T1DM cases from the south-east region of Romania were referred to our hospital both before and during the pandemic.

However, in the first 2 months of the pandemic, the number of new T1DM cases was lower than that reported in the same period of the previous year, the same difference being reported in another study which analyzed the number of children diagnosed with T1DM in Italy in the first 2 months of the pandemic [10]. In those first weeks of the pandemic, in our country a strict lockdown was imposed, with the population being allowed to leave the house only for a few clearly defined reasons like buying food and essential products, going to work in cases where the job was an essential one, and for mandatory medical assistance. Therefore, the lifestyle of children went through significant changes, one consequence being a significantly reduced exposure to common infectious microorganisms. Considering the potential of viral infections to act as triggers for the development of T1DM, the decreased incidence of this category of diseases might be an important cause of the lowered T1DM new cases at the beginning of the pandemic.

The physiopathological mechanisms that could play a role in the increase of the T1DM cases are yet to be completely understood but important progress in this direction was made by gathering evidence that SARS-CoV-2 is able to infect and replicate in pancreatic β-cells, leading to impaired function [13].

The children included in this study and diagnosed between March 2020 and February 2021 had a higher risk of presenting with DKA, time of presentation being significantly associated with increased likelihood of DKA diagnosis and higher DKA severity when adjusting for known predictors. While younger age, higher levels of HbA1C and presentation during the pandemic were significantly associated with a higher likelihood of being diagnosed with DKA, presentation during the pandemic was the only variable significantly associated with increased DKA severity. Previous studies found that the risk of developing DKA is higher in females, children with migration background, lower socio-economic status, children under 3 years old, early teenage years, patients without any first-degree relatives with T1DM and delayed diagnosis [18,19,20].

The addition of time of presentation to the multivariable explanatory model resulted in increased predictive performance, as reflected by a 2-fold increase in Nagelkerke’s pseudo-R^2^ for the DKA diagnosis model and a 4-fold increase for the DKA severity model. However, the absolute proportions of explained variability are relatively low even when including time of presentation, which indicates poor predictability of the outcomes given the variables we have considered. Although the purpose of this analysis is effect estimation rather than prediction, weak [18] predictive performance might indicate that other important predictors of outcome exist, which we did not consider in our analysis. The strong effect we have identified for time of presentation could be confounded by such an important unobserved predictor, and as such our results should be interpreted with caution.

An increase in DKA frequency and severity has been prior reported in other countries [7,8,9,10,11,12]. As DKA is a life-threatening condition that requires complex management, sometimes possible only in an intensive care unit, the increased frequency of this metabolic complication could lead to extensive pressure on an already overwhelmed healthcare system. On the other hand, pointing out that an increase in the incidence of a chronic pediatric disease might be one of the consequences of SARS-CoV-2 exposure might change the paradigm that children are not remarkably affected by the pandemic.

The duration of symptoms did not differ significantly during the pandemic compared to the previous years, this observation supporting the fact that an early diagnosis was made in these cases and being in contradiction with the hypotheses suggesting that the increase in the DKA frequency is caused by a delay in the diagnosis of T1DM [9,10]. A multicentre study performed in the United Kingdom also reported a short duration of symptoms in the majority of children [7].

Another interesting observation was that the mean age at diagnosis was higher in the pandemic group. Considering that it has long been recognized that DKA is more frequent in children of younger ages at the onset of T1DM [2], an increase in DKA frequency would be expected to associate a lower mean age of the patients.

One limitation of this study is represented by the fact that the pre-pandemic group included only the children that were under observation in our hospital during the evolution of T1DM and not all the cases diagnosed, while the pandemic group included all children diagnosed with T1DM in our hospital during the pandemic. This has been addressed in the effect estimation analysis for presence of DKA and DKA severity by using a propensity score matched cohort, in which every patient diagnosed in the pandemic period was matched to a patient diagnosed in the pre-pandemic period. In the matched cohort, the effect of presentation during the pandemic remained significantly associated with both higher likelihood of DKA at diagnosis and higher DKA severity. Another limitation is that the SARS-CoV-2 specific antibodies were not available for the new cases of T1DM. Additionally, the duration of symptoms might be insufficiently accurate as the information was gathered from the patients’ parents or guardians. The main strength of this study, on the other hand, is represented by the large sample used: a relatively high number of patients were included in the analysis. Moreover, all patients included in the study were diagnosed with T1DM in the same hospital and all the blood tests referenced were performed in the same laboratory, which ensures consistency.

As explained at the beginning of this paper, it is highly necessary to further investigate the relation between the increase in the number of cases and severity of T1DM cases in children and SARS-CoV-2 infection. More data supporting this link could be gathered by analyzing the presence of SARS-CoV-2 antibodies as evidence of previous infection in children newly diagnosed with T1DM.

## 5. Conclusions

Considering the fact that T1DM is one of the most frequent chronic pediatric illnesses and that its main acute complication, DKA is a life-threatening condition, it is important to analyze the repercussions that the new global pandemic might determine. Between March and February 2020, in Bucharest (Romania) and the surrounding areas there was a 30.08% increase in the total number of new T1DM cases in children. The patients that presented during the pandemic had a lower mean age, higher values of HbA1c, and were living more frequently in a rural area compared to the previous years. The number of diabetic ketoacidosis presentations also showed an increase during the pandemic, patients diagnosed after March 2020 having a significantly higher risk of developing DKA, when adjusting for other predictors. Moreover, a higher percentage of children presented a severe form of this metabolic complication. In the subgroup of patients that presented with DKA, the time of diagnosis (i.e., during the pandemic) was the only variable that showed a significant association with increased severity. These findings are supporting the idea that an increase in the incidence and severity of T1DM in children could be one of the consequences of COVID-19 pandemic. The duration of T1DM symptoms did not differ significantly between the pandemic and the pre-pandemic group; therefore, a delay in the presentation is unlikely to have been the main cause of the increased number of DKA presentations. Among the patients with the T1DM onset during the pandemic, 8 of them also presented SARS-CoV2 infection at the time of diagnosis.

Taking all these results into consideration we believe that further investigations are needed in order to discover other possible particular characteristics of T1DM cases diagnosed during the pandemic. Also, we are taking into consideration that a prospective cohort study including these patients could bring relevant additional data.

## Figures and Tables

**Table 1:** Characteristics of the study sample according to the time of diagnosis.

Group of Patients	Pre-Pandemic Group n (%)	Pandemic Group n (%)	*p* Values ^a^
Gender			
Male	170 (54.49)	75 (51.02)	
Female	142 (45.51)	72 (48.98	0.552
Background			
Urban	261(83.65)	91 (61.90)	
Rural	51 (16.35)	56 (38.10)	<0.001
Age of onset (median [IQR])	7.00 [3.00, 10.00]	7.20 [7.07, 7.30]	<0.001
Symptomatology duration (days)	25.38 ± 2.23	24.52 ± 1.83	0.51
HbA1c (%) (mean (SD))	11.32 ± 2.18	12.47 ± 2.19	<0.001
pH (median [IQR])	7.21 [7.12, 7.29]	7.20 [7.07, 7.30]	0.55

^a^*p* values correspond to χ^2^ statistics for categorical variables, Wilcoxon rank sum tests for non-normally distributed continuous variables (age, pH) and t-tests for normally distributed continuous variables (HbA1c), comparing the two subgroups. The *p* value assesses compatibility with the null hypothesis of no differences between subgroups.

**Table 2:** DKA presence and severity at the time of diagnosis in the pre-pandemic and pandemic group.

Group of Patients	Pre-Pandemic Group n (%)	Pandemic Group n (%)
No DKA	189 (60.58)	50 (34.01)
Total DKA	123 (39.42)	97 (65.99)
Severe DKA	33 (26.83)	41 (42.27)
Moderate DKA	36 (29.27)	24 (24.74)
Mild DKA	54 (43.90)	32 (32.99)

**Table 3:** Characteristics of the study sample, overall and according to DKA diagnosis.

	Overall	No DKA	DKA	*p* Value ^a^	Percentage of Missing Values
*n*	459	239	220		
Gender = M (%)	245 (53.4)	127 (53.1)	118 (53.6)	0.989	0
Age (median [IQR])	7.00 [4.00, 10.00]	8.00 [5.00, 11.00]	7.00 [3.00, 10.00]	0.006	0
Background = R (%)	106 (23.1)	48 (20.1)	58 (26.4)	0.138	0
HbA1c (mean (SD))	11.70 (2.25)	11.45 (2.27)	11.96 (2.19)	0.017	2.8
period = pandemic (%)	147 (32.0)	50 (20.9)	97 (44.1)	<0.001	0

^a^*p* values correspond to χ^2^ statistics for categorical variables, Wilcoxon rank sum tests for non-normally distributed continuous variables (age, pH) and t-tests for normally distributed continuous variables (HbA1c), comparing the two subgroups. The *p* value assesses compatibility with the null hypothesis of no differences between subgroups.

**Table 4:** Characteristics of the DKA subset, according to DKA severity.

	Mild DKA	Moderate DKA	Severe DKA	*p* Value ^a^	Percentage of Missing Values
N	85	61	74		
Gender = M (%)	46 (54.1)	34 (55.7)	38 (51.4)	0.873	0
Age (median [IQR])	6.00 [3.00, 10.00]	5.00 [2.00, 9.00]	8.00 [5.00, 10.00]	0.063	0
Background = R (%)	23 (27.1)	16 (26.2)	19 (25.7)	0.98	0
HbA1c (mean (SD))	12.10 (2.16)	11.44 (2.17)	12.22 (2.20)	0.086	0.9
period = pandemic (%)	32 (37.6)	24 (39.3)	41 (55.4)	0.054	0

^a^*p* values correspond to χ^2^ statistics for categorical variables, Kruskal-Wallis rank sum tests for non-normally distributed continuous variables (age, pH) and ANOVA for normally distributed continuous variables (HbA1c), comparing the three subgroups. The *p* value assesses compatibility with the null hypothesis of no differences between subgroups.

**Table 5:** Logistic regression of variables associated with DKA diagnosis.

Outcome: Positive DKA Diagnosis OR > 1 Mean Higher Likelihood of Outcome		Univariable Analysis OR (95% CI, *p*-Value)	Multivariable Analysis ^a^ OR (95% CI, *p*-Value)
Gender	F	Ref.	Ref.
	M	1.02 (0.71–1.47, *p* = 0.915)	1.08 (0.73–1.61, *p* = 0.686)
Age ^b^	Per year increase	0.94 (0.90–0.98, *p* = 0.005)	0.87 (0.82–0.92, *p* < 0.001)
Background	U	Ref.	Ref.
	R	1.42 (0.92–2.21, *p* = 0.112)	1.06 (0.65–1.72, *p* = 0.823)
HbA1c	Per percent increase	1.11 (1.02–1.21, *p* = 0.017)	1.17 (1.06–1.30, *p* = 0.002)
Period ^c^	Pre-pandemic	Ref.	Ref.
	Pandemic	2.98 (1.99–4.52, *p* < 0.001)	3.23 (2.06–5.15, *p* < 0.001)

^a^ Complete-case analysis: 446 patients included (13 excluded due to missing HbA1c data). ^b^ No significant non-linear relationships (modeled with splines) between the continuous covariates (Age, HbA1c) and outcome were detected. ^c^ Nagelkerke’s pseudo-R^2^ of the multivariable model increased from 0.08 to 0.15 when adding presentation time in the model.

**Table 6:** Proportional odds logistic regression of variables associated with increased DKA severity.

Outcome: Higher DKA Severity Class OR > 1 Mean Higher Likelihood of Outcome		Univariable Analysis OR (95% CI, *p*-Value)	Multivariable Analysis ^a^ OR (95% CI, *p*-Value)
Gender	F	Ref.	Ref.
	M	0.92 (0.56–1.5, *p* = 0.744)	0.97 (0.59–1.59, 0.897)
Age ^b^	Per year increase	1.04 (0.98–1.1, *p* = 0.166)	1.04 (0.97–1.11, 0.3)
Background	U	Ref.	Ref.
	R	0.95 (0.54–1.64, *p* = 0.842)	0.79 (0.44–1.4, 0.421)
HbA1c	Per percent increase	1.01 (0.91–1.13, *p* = 0.829)	0.94 (0.82–1.07, 0.364)
Period ^c^	Pre-pandemic	Ref.	Ref.
	Pandemic	1.75 (1.06–2.87, *p* = 0.028)	1.85 (1.07–3.23, 0.029)

^a^ Complete-case analysis: 218 observations included (two excluded due to missing HbA1c data). ^b^ No significant non-linear relationships (modeled with splines) between the continuous covariates (Age, HbA1c) and outcome were detected. ^c^ Nagelkerke’s pseudo-R^2^ of the multivariable model increased from 0.01 to 0.04 when adding presentation time in the model.

**Table 7:** Characteristics of the patients presenting SARS-CoV-2 inffection at the time of diagnosis of T1DM.

No.	Gender	Age	Days of Hospitalization	pH	HbA1c (%)	C Peptide (ng/mL)	CRP (mg/dL)	Vitamin D (ng/mL)
1	F	16	8	7.09	10.40			15.45
2	F	2	10	7.22	12.20	0.39	39.98	33.40
3	M	3	11	7.20	14.00	0.38	1.21	
4	M	12	6	7.13	12.70	0.11	1.21	15.95
5	F	1	12	7.13	10.70		0.36	64.27
6	F	17	17	6.99	11.20	0.7	2.72	
7	M	11	10	7.36	12.50		0.27	29.09
8	M	9	10	7.22	13.80		11	

## Data Availability

Not applicable.

## Supplementary Materials

The following are available online at https://www.mdpi.com/article/10.3390/jpm11060551/s1, Figure S1: title, Table S1: title, Video S1: title.
