# Supplementary figures and images for: The Impact of SARS-CoV-2 Pandemic on the New Cases of T1DM in Children. A Single-Centre Cohort Study
Source: J Pers Med. 2021 Jun 13;11(6):551. doi: 10.3390/jpm11060551 (PMC8231839; doi:10.3390/jpm11060551)

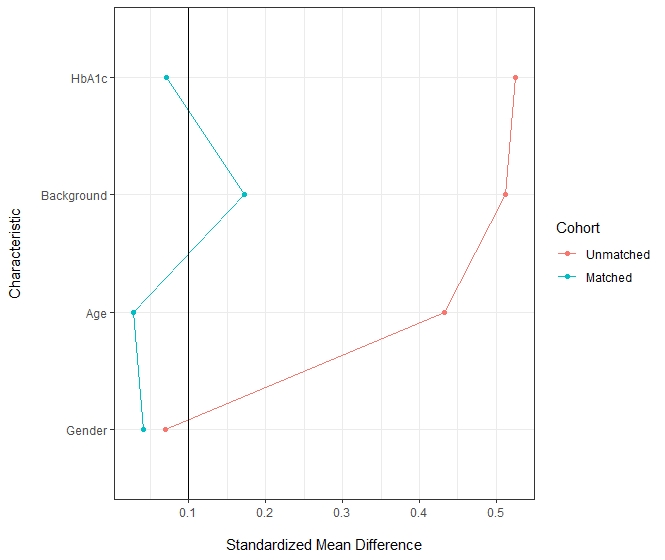

Supplement: Supplementary file 1 [file jpm-11-00551-s001.zip › Supplementary Figure 1.jpeg]
